# The delivery assessment for small targets on Halcyon radiotherapy system – Measured and calculated dose comparison

**DOI:** 10.1002/acm2.14407

**Published:** 2024-05-22

**Authors:** Linda Lankinen, Antti Kulmala, Jouko Lehtomäki, Ari Harju

**Affiliations:** ^1^ Varian Medical Systems, a Siemens Healthineers Company Helsinki Finland; ^2^ Department of Physics University of Helsinki Helsinki Finland; ^3^ Clinical Research Institute HUCH Ltd. Helsinki Finland

**Keywords:** Halcyon, Monte Carlo, stereotactic, small field, VMAT

## Abstract

**Background:**

With the ever‐increasing requirements of accuracy and personalization of radiotherapy treatments, stereotactic radiotherapy (SRT) with volumetric modulated arc therapy (VMAT) on O‐ring Halcyon radiotherapy system could potentially provide a fast, safe, and feasible treatment option.

**Purpose:**

The purpose of this study was to assess the delivery of Halcyon VMAT plans for small targets.

**Methods:**

Well‐defined VMAT‐SRT plans were created on Halcyon radiotherapy system with the stacked and staggered dual‐layer MLC design for the film measurement set‐up and the target sizes and shapes designed to emulate the targets of the stereotactic treatments. The planar dose distributions were acquired with film measurements and compared to a current clinical reference dose calculation with AcurosXB (v18.0, Varian Medical Systems) and to Monte Carlo simulations. With the collapsed arc versions of the VMAT‐SRT plans, the uncertainty in dose delivery due to the multileaf collimator (MLC) without the gantry rotation could be separated and analyzed.

**Results:**

The target size was mainly limited by the resolution originated from the design of the MLC leaves. The results of the collapsed arc versions of the plans show good consistency among measured, calculated, and simulated dose distributions. With the full VMAT plans, the agreement between calculated and simulated dose distributions was consistent with the collapsed arc versions. The measured dose distribution agreed with the calculated and simulated dose distributions within the target regions, but considerable local differences were observed in the margins of the target. The largest differences located in the steep gradient regions presumably originating from the deviation of the isocenter.

**Conclusions:**

The potential of the Halcyon radiotherapy system for VMAT‐SRT delivery was evaluated and the study revealed valuable insights on the machine characteristics with the delivery.

## 1 INTRODUCTION

Stereotactic radiotherapy (SRT) or stereotactic body radiotherapy (SBRT) is a high precision radiotherapy technique where the treatment is accurately focused on the small cancer target, reaching high effective dose with a few fractions, which results in a high effective dose.^1^ The goal of highly uniform coverage dose distributions including steep dose gradients must be achieved while minimizing healthy tissue irradiation. An accurate radiotherapy plan and delivery are required to ensure the sharp dose fall‐off outside the target to protect the surrounding healthy tissues. SRT with volumetric modulated arc therapy (VMAT) can offer an efficient delivery option with high‐dose conformality and lower toxicity complications.^2^, ^3^, ^4^, ^5^

Halcyon and Ethos (Varian Medical Systems, a Siemens Healthineers Company) treatment delivery systems must match with the demands of the dosimetric accuracy and robust patient immobilization/positioning so that the delivery would be suitable for VMAT‐SRT treatments. The design of the multileaf collimator (MLC) offers an effective resolution of 5 mm at the isocenter plane, which is similar to the standard Millennium 120 (5 mm) and double compared to High‐Definition (2.5 mm) MLCs found on TrueBeam/Edge linacs. Therefore, the beam shaping ability for a multiple‐target single‐isocenter setting is comparable to the standard Millennium 120 MLC^6^ and to High‐Definition MLC at least for targets with diameter > 1 cm.^7^ Additionally, the stacked and staggered dual‐layer design simultaneously minimizes the interleaf leakage and the transmission through the leaves. These design characteristics offer the potential to reduce dose to anatomy outside the target region as compared to millennium or High‐Definition MLC.

VMAT‐SRT is a complex treatment with multiple layers of uncertainties which needs to not only generate theoretically good treatment plans but also ensure that such plans can be delivered within the SRT accuracy requirements. The accuracy of the machine delivery, including mechanical accuracy of the gantry position, collimator position, and MLC position, needs to match to the demands of the treatment plans. The purpose of this work is to perform an end‐to‐end assessment of the performance and accuracy of Halcyon treatment delivery system with VMAT‐SRT deliveries. The output of this paper can be applied to both Halcyon and Ethos Radiotherapy Systems platforms as they share the same beam generation and collimation components. The system has proven to provide standardized and fast SRT treatments with curative intent and has demonstrated improved clinical workflow.^6^, ^8^, ^9^, ^10^

The aim is to collect high‐resolution measured dose distribution data with well‐defined VMAT‐SRT plans and compare the results to high‐accuracy Monte Carlo (MC) model^11^ and Acuros external beam (AXB) calculations.^12^, ^13^ AXB calculation was also included as it is the current clinical solution for the final dose calculation. With the measured and simulated reference dose distributions, the uncertainty related to the dose delivery of the system and the uncertainty related to dose calculation with AXB could be separated and analyzed. It should be noted that the plans are not clinically used but produced only for research purposes to quantify the agreement between the machine delivery and predicted dose distributions.

## 2 METHODS

### 2.1 Plans

**FIGURE 1:**
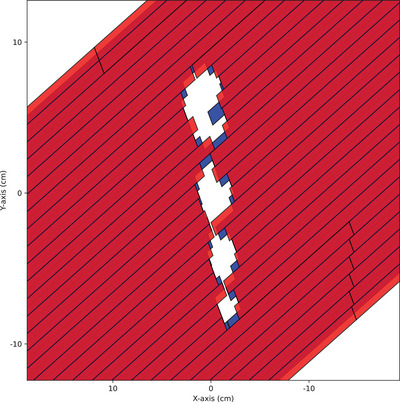
An example beams‐eye view of the MLC collimation in one control point. Distal, closer to the isocenter, layer leaves are shown in blue and proximal layer leaves are shown in red. MLC, multileaf collimators.

The treatment plans in this study are not clinically used but the target sizes and shapes mimic stereotactic treatments. The plans have been created so that the film measurements can be performed reliably with high accuracy. Measured, AXB calculated, and MC simulated dose distributions were obtained for plans with two optimized VMAT arcs (Photon Optimizer 18.0^14^) and one static alignment field with zero‐degree gantry, which was used for confirming the position of the film. Each plan had four spherical targets and the static alignment field had square 5 mm × 5 mm openings. The VMAT plans were separated into two categories with the diameters of spherical targets (4.0, 3.0, 2.0, 1.0) cm and (1.0, 0.8, 0.6, 0.4) cm. The target structures were concentric and aligned with a diagonal axis of the film. The objectives were set as in Table 1, and the nonspecified normal tissue had priority of 100. The same VMAT plans without the gantry rotation were measured as a quality assurance (QA) exclusively to investigate uncertainty in dose delivery and dose modeling due to the MLC. An example MLC collimation snapshot extracted from a VMAT plan is shown in Figure 1. The beam‐eyes view shows the four circular openings limited by the distal MLC leaves (blue, closer to the isocenter) and proximal MLC leaves (red).

**TABLE 1:** Objective settings for the full target structure and for the individual target spheres.

	Vol (%)	Dose (Gy)	Priority
Lower	100.0	2.96	100.0
Higher	100.0	3.04	100.0
Mean		3.00	100.0

### 2.2 Film measurements

Measurements were repeated with two clinically matching machines after approved by the daily machine performance check (MPC). Dose between two machines was averaged and used to minimize the variations of the machine‐specific uncertainties. An open field (10 cm × 28 cm) comparison was made to cross‐calibrate the daily variations of the actual machines, which were assumed to be minimal. Measured dose distributions were obtained by using GafChromic EBT3 radiochromic film (Ashland Speciality Ingredients G.P., Bridgewater, NJ), the Vidar DosimetryPro Advantage (red) densitometer (Vidar Systems Corporation, Herndon, VA), and the RW3 water‐equivalent plastic phantom (PTW, Freiburg, Germany). The measurement was repeated three times with both devices and each film was digitized three times. The measured dose distribution was aligned to the collimator coordinates using the alignment field. The phantom size was 30 cm × 30 cm × 10 cm and the film was set at 5 cm depth for all cases. Source to surface distance (SSD) was 95 cm. The machine type has fixed 800 MU/min dose rate.

To determine a high spatial resolution dose distribution in an environment where the photon energy spectrum is changing and unknown, three assumptions were made: (1) the radiochromic film is nearly water‐equivalent, (2) the amount of polymerization is almost independent of radiation energy and dose rate, and (3) polymerization is a highly local event allowing nearly no partial volume effect (a theoretical measurement resolution < 0.1 mm).^15^, ^16^, ^17^ The accuracy of the film dosimetry depends highly on proper film handling, suitable development protocols, and calibrated densitometry system.^18^, ^19^ High dosimetry standards have been applied, for example, a session‐specific optical density to dose calibration has been defined and a densitometer spatial response correction has been determined.

The experimental uncertainties included the spatial uncertainty, the uncertainty of absolute dose calibration, and the random uncertainty/'pixel to pixel noise'. The spatial uncertainty originates from the uncertainty of the orientation and alignment of the radiation beam. In film dosimetry, the systematic uncertainties are corrected by the restricted scanning procedure and scanner response correction and random uncertainties are avoided with repeated measurements. The use of an alignment field in the treatment plan aims to eliminate the uncertainties in the film positioning. The combined measurement uncertainty was estimated to be less than 3%.

### 2.3 AXB calculations

In general, individual delivery systems are within good consistency to the vendor provided preconfigured beam model.^20^ As Halcyon and Ethos Radiotherapy Systems share the same beam generation and collimation components, the platforms have identical preconfigured beam data and AXB algorithm can be used in both to generate final dose calculations. In this study, AXB calculations (v18.0) were performed using preconfigured beam data and dose‐to‐medium reporting. AXB v18.0 has performance‐driven approximation in the collimator system with the enhanced MLC model aims to model the true leaf tip geometry and has improved on‐axis and off‐axis agreements with small MLC fields.^21^ The calculation resolution of 1 mm was used.

### 2.4 MC simulations

The MC model can be used as a computational copy of an actual machine and provide reference without uncertainties due to the known mechanical variations, for example, in the MLC leaf positions. Our MC model was validated to match with the preconfigured beam data and the CAD implementation provided an accurate geometry model for the critical collimating components, especially, the MLC leaf tip regions. The MC simulations were performed as previously described in the reference.^11^ The particles were transported through the treatment head and into a water phantom where the dose deposit was recorded. The dose deposit for each field in each plan, two VMAT arcs and the static alignment field, were simulated separately and combined afterwards.

In the MC simulations, Geant4 physics list “QGSP_BIC” with “EM Opt4” was used. A resolution of 0.5 mm was selected, and the size of the water phantom set to match with the size of measured set up (30 cm × 30 cm × 10 cm). The reference geometry for the absolute dose calibration was set to 1 cGy per monitor unit (MU) at the depth of maximum dose with field size 10 × 10 ${\mathrm{cm}}^{2}$ and SSD of 100 cm.^22^ The mechanical deviation between planned and actual MLC leaf gaps was considered with the machine‐specific mechanical leaf gap (MLG). The MLG of ‐0.65 mm was used for both layers in nominal positions.

The statistical nature of the Monte Carlo method always includes uncertainties. The uncertainties were evaluated by the standard error of the mean. The square root of the number of simulated particles is inversely proportional to the relation between the uncertainty of dose and the dose. The uncertainty was estimated to be less than 2%.

### 2.5 Dose comparison

The dose planes at 5 cm depth with SSD 95 cm in virtual and physical water phantoms were calculated, simulated, and measured with the film. The measured dose distribution data described the actual output of the machine and the delivery accuracy was assessed by comparing the results to MC simulations and AXB calculations. AXB calculation provided a current clinical reference solution and MC simulation provided an independent reference benchmarked for the preconfigured beam data used in AXB calculations. The dose planes between the three methods were resampled to 1 mm resolution and then the dose differences were calculated. The global gamma passing rates for the dose planes excluding the alignment openings in comparison to the clinical reference, AXB calculation, were determined. An in‐house gamma calculator with criteria 2%/1 mm and a low‐dose cut‐off threshold of 10% was used.

## 3 RESULTS

### 3.1 Quality assurance plans without the gantry rotation

With the open field (10 cm × 28 cm), MC and AXB dose levels were consistent, but the averaged measured dose was 1.5% lower. As the difference is acceptable within the limits of the daily variations, the measured dose was scaled accordingly. The machine variations and daily fluctuation were not considered in either MC simulation or AXB calculation.

**FIGURE 2:**
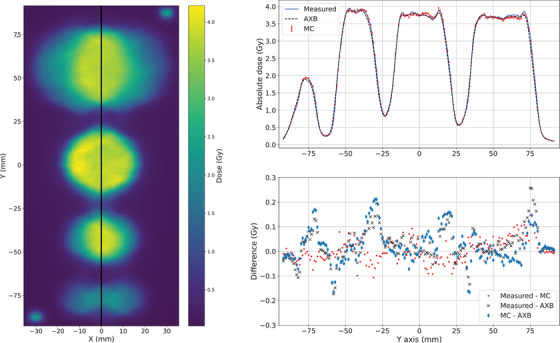
The measured dose plane on the left and on the right the dose profiles with local difference at X = 0 mm of the measured, AXB, and MC with collapsed arc versions of the VMAT plan and the conventional size (4.0, 3.0, 2.0, 1.0) cm targets. AXB, acuros external beam; MC, Monte Carlo; VMAT, volumetric modulated arc therapy.

First, VMAT plans without the gantry rotation were analyzed. These were collapsed arc versions of the VMAT plans, where the gantry was at the head‐up direction for all control points. The plans exclusively demonstrate the performance of the MLC in dose delivery and dose modeling. In Figure 2, the measured dose plane is presented on the left side and the profiles with the dose difference are presented on the right side with the conventional size (4.0, 3.0, 2.0, 1.0) cm diameter targets. The black line in the middle of the dose plane demonstrates the position of the profiles. The profiles at central line (X = 0 mm) and pointwise differences between measured (blue line), AXB (black dashed line), and MC (red markers) are presented. The absolute level of the AXB and MC dose agree with the film measurements and no major differences are shown. The local differences between measured, AXB, and MC are below 6%. The overall local differences between the measured and MC are smaller than between the measured and AXB. As shown, AXB deviates more with a slight underdosing on right sides of the peaks.

**FIGURE 3:**
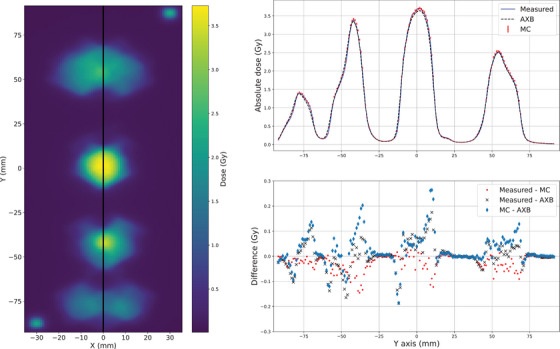
The measured dose plane on the left and on the right the dose profiles with local difference at X = 0 mm of the measured, AXB, and MC with collapsed arc versions of the VMAT plan and the small (1.0, 0.8, 0.6, 0.4) cm targets. AXB, acuros external beam; MC, Monte Carlo; VMAT, volumetric modulated arc therapy.

Similarly for the small (1.0, 0.8, 0.6, 0.4) cm diameter targets, the measured dose plane and the profiles with the dose difference are presented in Figure 3. The dose level is smaller than with the conventional size targets but the percentage differences are of the same order of magnitude. Minimal deviations are observed due the noise in the measured and MC data.

**FIGURE 4:**
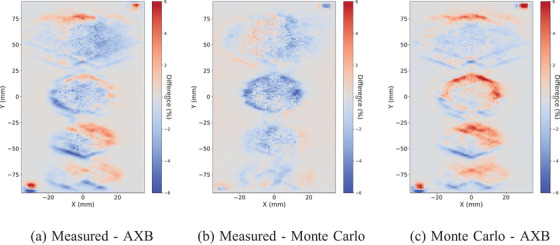
The voxelwise dose plane differences between the measured, AXB, and MC with collapsed arc versions of the VMAT plan and the conventional size (4.0, 3.0, 2.0, 1.0) cm targets. The maximum local difference is within 6%. AXB, acuros external beam; MC, Monte Carlo; VMAT, volumetric modulated arc therapy.

**FIGURE 5:**
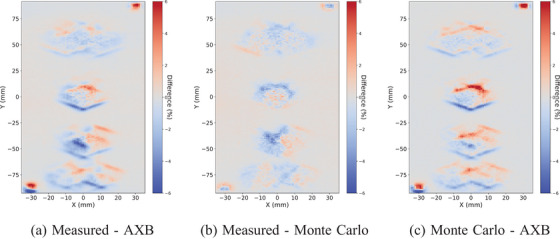
The voxelwise dose plane differences between the measured, AXB, and MC with collapsed arc versions of the VMAT plan and the small (1.0, 0.8, 0.6, 0.4) cm targets. The maximum local difference is within 6%. AXB, acuros external beam; MC, Monte Carlo; VMAT, volumetric modulated arc therapy.

The same observations are confirmed with the voxelwise comparisons between the measured, AXB, and MC dose planes with the conventional size targets in Figure 4 and with the small targets in Figure 5. The comparison between the measured and AXB is on the left, between the measured and MC on the center, and between MC and AXB on the right. In all, the overall local agreement is within 6%. The deviations between the measured and MC are smaller than in comparison to AXB. At the edges of the dose distributions, similar differences between measured and AXB and between MC and AXB are observed. The largest differences are between MC and AXB, and especially, in the alignment fields with 5 × 5 mm openings, where AXB underestimates the dose.

### 3.2 VMAT plans with the gantry rotation

**FIGURE 6:**
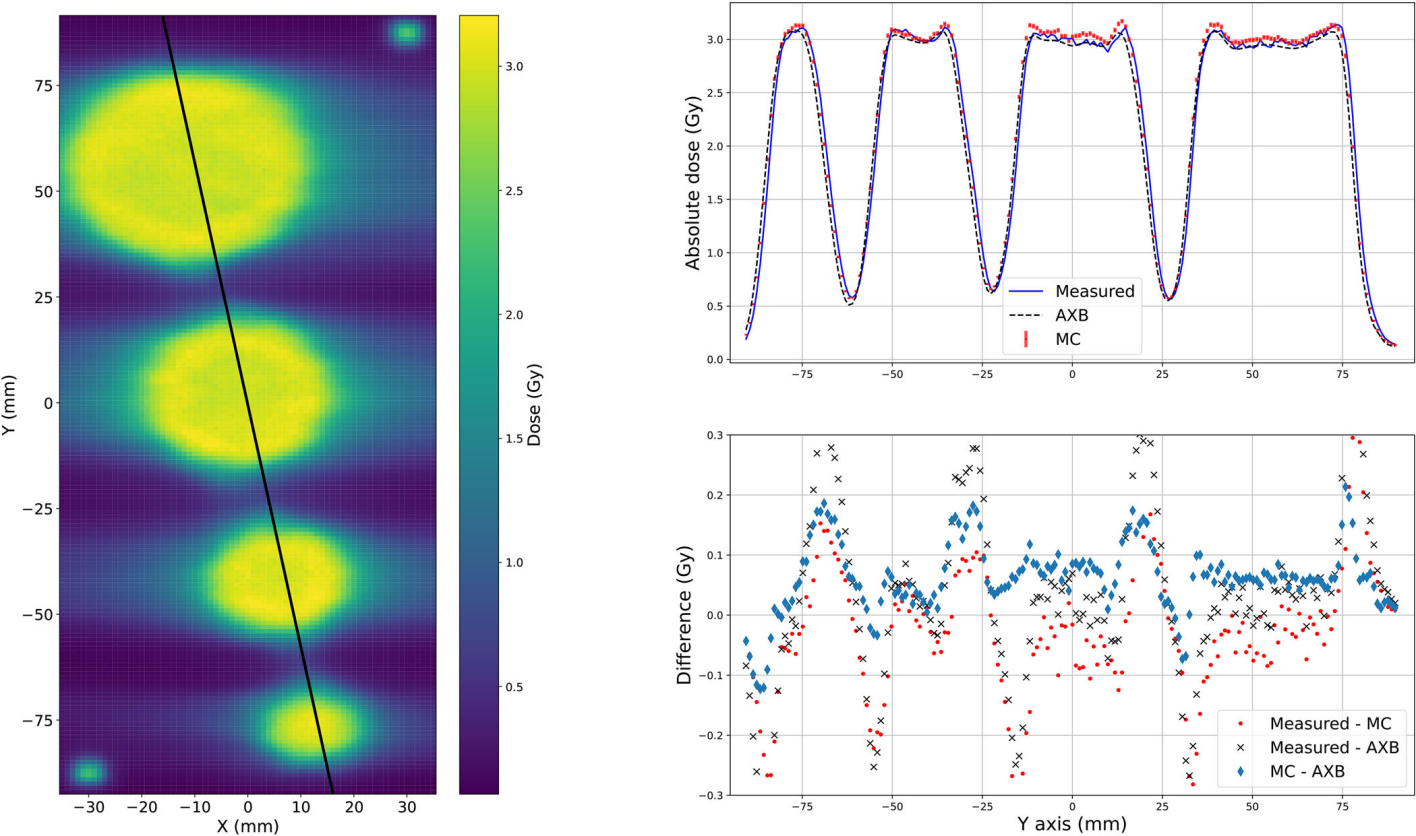
The measured dose plane on the left and on the right the dose profiles with local difference of the measured, AXB, and MC with VMAT plan and the conventional size (4.0, 3.0, 2.0, 1.0) cm targets. AXB, acuros external beam; MC, Monte Carlo; VMAT, volumetric modulated arc therapy.

Next, the VMAT plans were studied. The goal of 3 Gy in all targets was reached with both the conventional‐ and small‐sized targets. In Figure 6, the measured dose plane is presented on the left side and the profiles with the dose difference are presented on the right side with the conventional size (4.0, 3.0, 2.0, 1.0) cm targets. As demonstrated with black line, the profiles are taken so that they pass through each spherical target. A systematic shift with measured dose is shown in the profiles with the measured (blue line), AXB (black dashed line), and MC (red markers). Although, the measured dose level inside the targets matches with both AXB and MC, at the edges of each spherical targets, the local deviations of up to 10% are observed. The shift seems to be more profound in comparison to AXB as the differences are the same order of magnitude on both sides of the target spheres. In comparison to MC, the differences are smaller on the right side of the spheres except with the largest sphere.

**FIGURE 7:**
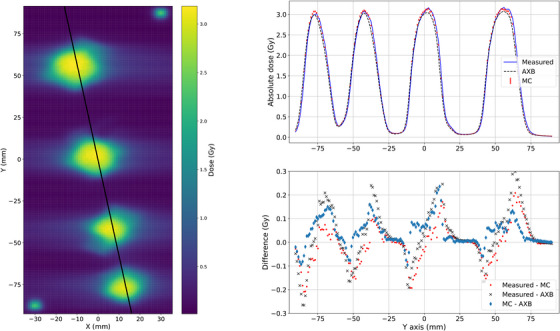
The measured dose plane on the left and on the right the dose profiles with local difference of the measured, AXB, and MC with VMAT plan and the small (1.0, 0.8, 0.6, 0.4) cm targets. AXB, acuros external beam; MC, Monte Carlo; VMAT, volumetric modulated arc therapy.

The same systematic shift is also shown in the profiles with the small (1.0, 0.8, 0.6, 0.4) cm targets in Figure 7. With both target sizes, the shift with measured dose is roughly 0.5 mm in Y‐direction and creates the largest differences in the edges of the target spheres. Again with the small targets, the differences between the measured and AXB are the same order of magnitude on both sides of the spheres, but smaller between the measured and MC on the right side of the spheres. The dose level of measured seem to match better with MC than AXB with all spheres except with the smallest one.

**FIGURE 8:**
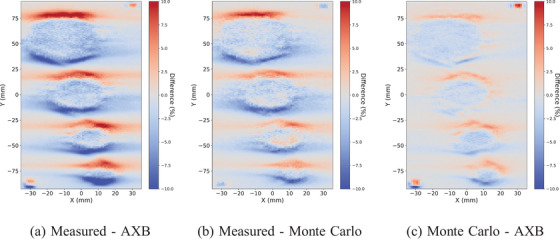
The voxelwise dose plane differences between the measured, AXB, and MC with VMAT plan and the conventional size (4.0, 3.0, 2.0, 1.0) cm targets. The maximum local difference is within 10%. AXB, acuros external beam; MC, Monte Carlo; VMAT, volumetric modulated arc therapy.

**FIGURE 9:**
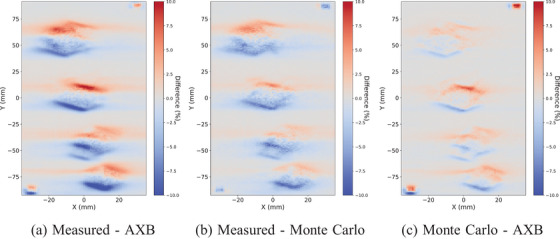
The voxelwise dose plane differences between the measured, AXB, and MC with VMAT plan and the small (1.0, 0.8, 0.6, 0.4) cm targets. The maximum local difference is within 10%. AXB, acuros external beam; MC, Monte Carlo; VMAT, volumetric modulated arc therapy.

The shift is obvious also in the voxelwise dose plane comparisons between the measured, AXB, and MC with the conventional size in Figure 8 and with the small targets in Figure 9. However, the alignment fields with gantry zero and 5 mm × 5 mm openings show no systematic differences, and therefore, the shift is not due to the positioning uncertainties. The largest dose differences reach up to 10% between the measured and AXB in the high‐dose gradient regions. The differences between the measured and MC are similar but slightly smaller with both target sizes. Between MC and AXB, some deviations at the edges of the targets are observed, but the largest differences are shown with the alignment fields.

### 3.3 Gamma passing rates

In Table 2, the gamma passing rates (GPR) obtained for the dose plane excluding the alignment openings with 2%/1 mm criteria and a low‐dose cut‐off threshold of 10% in comparison with AXB. For all dose distributions, GPRs of MC were above 98.9% and all the values were higher than with measured. The lowest GPR of 91.7% between measured and AXB dose distribution was obtained for the VMAT plan with the conventional size targets.

**TABLE 2:** Gamma passing rates (%) for the dose plane excluding the alignment openings with 2%/1 mm criteria and a low‐dose cut‐off threshold of 10% in comparison to AXB.

	MC	Measured
Collapsed arc with the conventional size targets	100.0	98.3
Collapsed arc with the small targets	100.0	100.0
VMAT with the conventional size targets	98.9	91.7
VMAT with the small targets	99.9	98.8

Abbreviations: AXB, acuros external beam; MC, Monte Carlo; VMAT, volumetric modulated arc therapy.

## 4 DISCUSSION

The purpose of the study was to evaluate the delivery accuracy of Halcyon radiotherapy system with dual‐layer stacked and staggered MLC on small‐target dosimetry. As the effective resolution is limited to 5 mm at the isocenter plane due to the design of the MLC leaf width, this study shows acceptable dose conformity with the target sizes up to 5 mm.

The collapsed arc versions of the VMAT plans show agreement between the measured, AXB with enhanced MLC model, and MC. The results confirm that the MLC delivery match with the calculated and the simulated data, and systematic differences due the mechanical uncertainties of the MLC were not observed. The local differences between MC and the measurements were smaller than between AXB and measurements as MC model has extremely detailed description of the MLC leaves, and does not use performance‐driven approximation in the collimator system that AXB needs to have to suit clinical use. The dose could be even closer with an improved model with interleaf leakage between two adjacent MLC leaves. With both the collapsed arc versions and the full VMAT plans, the largest differences between the calculated and the simulated data were seen with the alignment fields, which is only used for positioning and is not in the interest of this study. The differences are shown as the small static openings highlight the details of MLC modeling.

When the gantry rotation was included with VMAT plans, the match between AXB and MC show similar differences in the target regions as with the collapsed arc versions. In comparison to the measurements, differences up to 10% in high‐dose gradient regions within the CTV‐PTV safety margin (= 1 mm) were observed. As similar deviations were not observed between MC and AXB or in the collapsed arc versions of the VMAT plans, the deviation seems to be due to the mechanical uncertainty of the system as the gantry is rotating. The shift could be explained by small changes in the center of the gantry rotation relative to the treatment isocenter. The gravity pull on the beam centerline and the mechanical uncertainties cause systematic deviations of the isocenter position as a function of gantry angle.^23^ The radiation field centers “wobble” during gantry rotation and the component along the couch's longitudinal direction is referred to as gantry sag. Depending on the linac and the collimator angle, the measured values range between 0.3 and 1 mm for C‐arm linacs are reported,^23^, ^24^, ^25^ but Halcyon or O‐ring shaped system results have not been published. The systematic shift found in this study is close to these values and when combined with the GPR evaluation, indicates that the studied system is on par with the 1.00 mm and 1.5 mm radiation isocentricity for SRS and SBRT recommended by Halvorsen et al.^26^ Furthermore, in the AAPM Task Group Report 142 recommendation describing the linear accelerator QA for SRT procedures, the geometric position of the radiation isocenter during rotation is usually assumed to be inside a virtual spherical volume and up to ± 1 mm deviation is acceptable for stereotactic treatments.^27^ Moreover, the overall mechanical performance of the treatment system needs to meet the regulations set for SRT/SBRT accuracy.^28^ In this study, the isocenter position is commissioned at gantry angle zero, as such the effect is not visible in the collapsed arc versions of the VMAT plans. The role of gravity on the positional accuracy of MLC was disregarded in both AXB and MC and future research is needed to establish better understanding of the uncertainties.

GPRs were above 98.3% for both MC and measured except the VMAT plan with the conventional size targets, where GPR of the measured was 91.7%. The lowest GPRs were observed with the conventional targets as the edges of the targets introduced more gradient region. The tighter criterion of 2%/1 mm with a low‐dose cut‐off threshold of 10% was selected due to the position accuracy of the film measurements, and the SRT technique's use of high‐dose gradients and small margins.

In SRT‐VMAT deliveries with Halcyon, the gantry sag may become significant and should be taken in account, for example, with the margin of the target. Although, the gantry sag is a relatively small uncertainty compared to target delineation, patient setup error, and target movement. To ensure accurate treatment delivery, the long‐term mechanical stability of the system will also need to be well characterized, including Winston‐Lutz or similar tests and MLC positioning accuracy as a function of gantry angle for both layers. The clinical impact of mechanical uncertainty on VMAT‐SRT delivery warrants future studies.

## 5 CONCLUSIONS

The delivery accuracy of the Halcyon system equipped with dual‐layer MLC for VMAT stereotactic radiotherapy treatment was evaluated. Dosimetrically high‐quality dose measurement data was acquired and compared to dose calculation with AXB (v 18.0, Varian Medical Systems) and Monte Carlo simulations. The utility of the system for VMAT‐SRT delivery was assessed and the results provided more understanding about the mechanical uncertainties.

## CONFLICT OF INTEREST STATEMENT

The authors declare no conflicts of interest.
